# The Invested in Diabetes Study Protocol: a cluster randomized pragmatic trial comparing standardized and patient-driven diabetes shared medical appointments

**DOI:** 10.1186/s13063-019-3938-7

**Published:** 2020-01-10

**Authors:** Bethany M. Kwan, L. Miriam Dickinson, Russell E. Glasgow, Martha Sajatovic, Mark Gritz, Jodi Summers Holtrop, Don E. Nease, Natalie Ritchie, Andrea Nederveld, Dennis Gurfinkel, Jeanette A. Waxmonsky

**Affiliations:** 1 University of Colorado School of Medicine, 13199 E Montview Blvd Ste 210, Aurora, CO 80045 USA; 2 VA Eastern Colorado QUERI and Geriatric Research Centers, 1055 Clermont St, Denver, CO 80220 USA; 3 Case Western Reserve University, 10900 Euclid Ave, Cleveland, OH 44106 USA; 4 Denver Health and Hospital Authority, 777 Bannock St, Denver, CO 80204 USA

**Keywords:** Diabetes, Shared medical appointments, Diabetes self-management, Peer mentors, Cluster randomized pragmatic trial, Diabetes distress, Mixed methods, RE-AIM, Replicating effective programs, Implementation

## Abstract

**Background:**

Shared medical appointments (SMAs) have been shown to be an efficient and effective strategy for providing diabetes self-management education and self-management support. SMA features vary and it is not known which features are most effective for different patients and practice settings. The Invested in Diabetes study tests the comparative effectiveness of SMAs with and without multidisciplinary care teams and patient topic choice for improving patient-centered and clinical outcomes related to diabetes.

**Methods:**

This study compares the effectiveness of two SMA approaches using the Targeted Training for Illness Management (TTIM) curriculum. Standardized SMAs are led by a health educator with a set order of TTIM topics. Patient-driven SMAs are delivered collaboratively by a multidisciplinary care team (health educator, medical provider, behavioral health provider, and a peer mentor); patients select the order and emphasis on TTIM topics. Invested in Diabetes is a cluster randomized pragmatic trial involving approximately 1440 adult patients with type 2 diabetes. Twenty primary care practices will be randomly assigned to either standardized or patient-driven SMAs. A mixed-methods evaluation will include quantitative (practice- and patient-level data) and qualitative (practice and patient interviews, observation) components. The primary patient-centered outcome is diabetes distress. Secondary outcomes include autonomy support, self-management behaviors, clinical outcomes, patient reach, and practice-level value and sustainability.

**Discussion:**

Practice and patient stakeholder input guided protocol development for this pragmatic trial comparing SMA approaches. Implementation strategies from the enhanced Replicating Effective Programs framework will help ensure practices maintain fidelity to intervention protocols while tailoring workflows to their settings. Invested in Diabetes will contribute to the literature on chronic illness management and implementation science using the RE-AIM model.

**Trial registration:**

ClinicalTrials.gov, NCT03590041. Registered on 5 July 2018.

## Background

Diabetes is among the most prevalent chronic diseases in the United States, with estimates suggesting 12.2% of adults have diagnosed or undiagnosed diabetes [1]. Type 2 diabetes mellitus (T2DM) is the most common form of diabetes among adults [2]. Diabetes can be controlled with appropriate diet and physical activity as well as oral and injectable medications, yet as many as 49% of adults with diabetes do not meet targets for glycemic control [3]. Poorly controlled diabetes is associated with poor health outcomes, including neuropathy, retinopathy, nephropathy, cardiovascular disease, and premature death [4]. Despite recent decreases in rates of certain complications, diabetes remains a considerable source of disability and cost to the healthcare system [5]. The burden of diabetes is great**,** both in terms of patient out-of-pocket healthcare costs [6] and poor quality of life, especially among those with complications [7].

Patients with T2DM must engage in daily self-management activities including blood glucose monitoring, following dietary recommendations, getting regular physical activity, and adhering to prescribed medications (including insulin management in those who are insulin-dependent). Diabetes self-management is challenging, especially among low-income populations [8], and many patients experience diabetes distress, the sense of being overwhelmed with managing diabetes [9]. Diabetes distress stems from the regimen, interpersonal, emotional, and healthcare navigation burden associated with managing diabetes, and interferes with self-care and glycemic control [10].

According to the American Diabetes Association’s 2015 position statement, care for patients with T2DM should include antiglycemic therapy and cardiovascular risk reduction through weight loss, blood pressure reduction, and smoking cessation [11]. Wagner’s chronic care model (CCM) has informed how care should be delivered for patients with T2DM to help achieve these goals [12–14]. The CCM emphasizes whole-person care by addressing physical, mental health, and psychosocial needs [15]. Evidence shows patients with diabetes benefit from CCM-based approaches in primary care [16], including comprehensive diabetes self-management education (DSME) and self-management support (SMS) [17, 18]. Notably, SMS can decrease the burden of diabetes and improve diabetes distress [19].

Shared medical appointments (SMAs) can help practices efficiently and effectively provide DSME and SMS consistent with the CCM [20]. SMAs are “groups of patients meeting over time for comprehensive care, usually involving a practitioner with prescribing privileges, for a defining chronic condition or health care state” [21]. A 2014 systematic review and meta-analysis showed diabetes SMAs lead to significantly greater improvements in glycated hemoglobin (HbA1c) and blood pressure compared to usual care [21]. However, there was heterogeneity in these effects, suggesting some SMA models may be more effective than others. SMAs can vary in terms of the curriculum used, professional background of group facilitators, the frequency, duration, and number of group sessions, the number and types of patients involved, whether it is a closed or open group (same patients each time or patients can come and go from the group), involvement of family members, involvement of diabetes peer mentors, and topic selection (set order and emphasis on topics or flexible topic selection in which patients pick from a menu).

A key conclusion of the systematic review was the lack of evidence for which SMA features are most effective for improving outcomes important to patients and practices. Upon engaging primary care practices, diabetes patients and family members, and representatives from community health organizations in research prioritization [22], our stakeholders endorsed testing professionally led group visits using a curriculum that addressed both physical and mental health aspects of managing diabetes. The stakeholders wanted evidence on several key SMA features, including the relative value of behavioral health providers as members of a multidisciplinary care team delivering SMAs, standardizing educational topics versus supporting patient choice of topics and topic order, and including diabetes peer mentors [22] to support patients during and outside of group sessions. The Invested in Diabetes study was designed to test comparative effectiveness of diabetes SMAs with and without these key features.

The purpose of the Invested in Diabetes study is to compare effectiveness of two diabetes SMA models varying in these key features (multidisciplinary care teams, peer support, and flexible topic emphasis and order): standardized and patient-driven SMAs.

### Intervention conceptual model

**Fig. 1:**
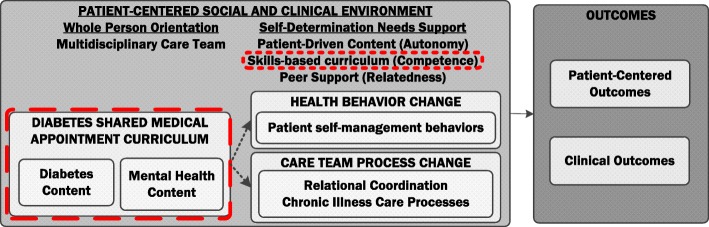
Invested in Diabetes study conceptual model

This study adheres to the SPIRIT guidelines for reporting clinical trials (SPIRIT Checklist, Additional file 1). The conceptual model (Fig. 1) underlying the distinction between comparator SMA models is based on self-determination theory (SDT) [23] and principles of whole-person care [24]. According to SDT, human motivation and behavior are a function of the social environment and the extent to which that environment supports basic psychological needs enhancing “self-determined motivation.” Considerable evidence supports SDT; studies show people tend to be more motivated to engage in an intervention and change their behavior when the intervention supports the need for autonomy (respect for choice and preference), competence (building self-efficacy, recognizing capacity for change), and relatedness (sense of belonging, understanding an individual’s values) [25, 26]. Key factors of SDT have been found to mediate improvement in outcomes in diabetes self-management studies [27, 28].

Both SMA models will use a curriculum with both diabetes and mental health content, reflecting a whole-person orientation. To support competence, the curriculum emphasizes building skills (e.g. problem solving, goal setting, communication skills) and enhancing self-efficacy. In the standardized SMA model, a single healthcare team member will deliver this curriculum following a set topic order, with a set amount of time to cover each topic. To further emphasize a whole-person orientation to care, in the patient-driven model of SMAs, the curriculum will be delivered by a multidisciplinary care team, including health educators, behavioral health providers, and peer mentors.

To enhance autonomy, patients in the patient-driven arm will select the topics they want and need at that particular point in time (i.e. choose the topics and the order in which they are presented). To support relatedness, the patient-driven model of SMA is co-facilitated by diabetes peer mentors, who are also available to patients outside of the group visit setting for individual meetings. The distinguishing features between the patient-driven and standardized models of SMA—multidisciplinary care team with peer support and patient-driven content—are the elements that represent a more patient-centered social and clinical context. These needs-supportive elements of the patient-driven approach may enhance self-determined motivation and help overcome barriers to diabetes self-management behaviors, thereby enhancing self-management resulting in improved glycemic control and patient-centered outcomes such as diabetes distress.

### Aims and hypotheses

The study aims for Invested in Diabetes are to:

1. Compare the reach and effectiveness of standardized versus patient-driven diabetes SMAs, for improving patient-centered outcomes (diabetes distress [the primary outcome for this study, a patient-reported outcome], perceived autonomy support, diabetes self-care behaviors), patient clinical outcomes (HbA1c, blood pressure, and body mass index [BMI]), patient acceptance and attendance at SMAs, and practice-level outcomes (quality of diabetes care and relational coordination); and
2. Describe factors associated with practice adoption, implementation, and maintenance of standardized and patient-driven diabetes SMAs, including resource requirements and costs to practices and patients (out-of-pocket cost and time commitment).

Compared to standardized diabetes SMAs, we hypothesize patients participating in patient-driven diabetes SMAs will report greater improvements in patient-centered outcomes, including diabetes distress (primary outcome), autonomy support, quality of life, and diabetes self-management behaviors (secondary) (Hypothesis I) and in HbA1c, blood pressure, and BMI (Hypothesis II). Among eligible patients agreeing to participate in SMAs, those offered the patient-driven model will attend more scheduled sessions than those offered the standardized model (Hypothesis III). Compared to standardized diabetes SMAs, we hypothesize practices using patient-driven diabetes SMAs will exhibit greater improvements in quality of care and team-based care (Hypothesis IV).

## Methods

### Trial design

Invested in Diabetes is a cluster randomized pragmatic trial, with randomization clustered at the practice level using covariate-constrained randomization [29–31]. Twenty primary care practices will be randomly assigned to either standardized or patient-driven diabetes SMAs (10 per condition; Table 1). During the 24-month implementation period, each practice will conduct SMAs with at least eight cohorts of approximately 8–10 patients each (ultimately yielding at least 72 patients per practice; 60 patients with complete data). Cohorts of adults with T2DM will complete six SMA sessions as a closed group. A mixed-methods evaluation will include quantitative (practice and patient-level surveys, electronic health record [EHR] data, and patient participation) and qualitative (practice and patient interviews, intervention fidelity and adaptations observation) components.

**Table 1:** Distinguishing features between standardized and patient-driven diabetes SMAs for Invested in Diabetes

	Arm 1: Standardized SMAs	Arm 2: Patient-driven SMAs
Same for both arms		
No. and duration of sessions	Six 2-h group sessions with 8–10 adult patients with T2DM	
Educational components	Diabetes and mental health with goal setting and psychosocial support topics using the TTIM curriculum	
SMA coordinator role	Patient identification, recruitment, reminders, care team scheduling, and clinical documentation	
Prescribing provider role	Patients step out of group for brief visits with provider with prescribing privileges for medication management and patient-specific medical advice	
Distinguishing features		
Patient topic choice	Order of and time spent on TTIM topics are set for all SMA cohorts	Patients in each SMA cohort select order of and time spent on TTIM topics
Health educator role	Lead instructor for all educational components	Co-facilitator with peer mentor for non-mental health topics
Behavioral health provider role	Not involved in SMAs	Co-facilitator with peer mentor for mental and behavioral health topics
Peer mentor role	Not involved in SMAs	Co-facilitator for all group visits; 1 × 1 peer access

*SMA* shared medical appointments, *T2DM* type 2 diabetes mellitus, *TTIM* Targeted Training in Illness Management

### Study setting

The setting for this study is primary care practices including Federally Qualified Health Centers (FQHCs) serving primarily public payer populations and private/health system-affiliated practices serving primarily commercial payer populations. To participate, practices must have: (1) a current panel of at least 150 adult patients with T2DM; (2) access to health educators, integrated behavioral health providers, and diabetes peer mentors; and (3) willingness to be randomly assigned to implement either patient-driven or standardized SMAs.

### Participant eligibility criteria

Participating patients must: (1) be aged at least 18 years; (2) have T2DM; (3) speak English or Spanish; and (4) receive care in a participating practice. Patients will be excluded from the primary analysis if they are pregnant during the study period, have limited cognitive ability due to dementia or a developmental disorder, or have a diagnosis with less than one year of life expectancy.

### Interventions

#### General structure of SMAs

SMA features of the intervention arms—including those features that are the same versus vary across arms—are described in Table 1. Both intervention arms use the same curriculum to deliver six sessions of 2-h SMAs to groups of 8–10 patients with T2DM, with groups meeting weekly, bi-weekly, or monthly according to practice preference. Patients step out of group for brief (5–10 minutes) individual visits with a provider with prescribing privileges, who provides medication management, orders and referrals, and patient-specific medical advice. Curriculum, dose of intervention, frequency of sessions, visits with medical providers, and group size are consistent across study arms, and are thus not variables in the study. Each practice also designates someone to serve as an SMA coordinator, to support recruitment, scheduling, and follow-up with patients participating in SMAs.

#### Curriculum

Targeted Training for Illness Management (TTIM) is an evidence-based, manualized, modular group intervention for the self-management of chronic illness, originally developed for people with severe mental illness. The evidence-based TTIM approach has been successfully adapted for a variety of chronic health conditions including epilepsy, stroke, Parkinson’s disease, and diabetes [32–36]. TTIM was selected for this study by our stakeholders because it met the criteria for having both physical and mental health topics and had a version specific to diabetes. The Invested in Diabetes team adapted the diabetes version of TTIM for this study context based on stakeholder input, as part of the implementation framework described below. Adaptations included reorganizing content to fit within six sessions of 2 h rather than 12 sessions of 1 h, adding content on general stress and coping skills to supplement content focused on mental illness management alone, and updating nutrition and physical activity content based on recent evidence and guidelines. The TTIM modules and when they are covered in each study arm are listed in Table 2. Module 4 has two versions, one for a general population of patients with T2DM (stress and coping content) and one for a population of patients with T2DM and co-occurring mental illness (mental illness and coping content). Practices choose whichever version fits their patient population. The TTIM manual includes instructions and scripts for group facilitators, patient handouts and home exercises, and visuals that can be projected on a monitor.

**Table 2:** TTIM modules for standardized and patient-driven SMAs

TTIM module topic	Standardized SMAs	Patient-driven SMAs
Module 1: Setting the Stage and Introduction to Diabetes, Baseline Patient-Reported Outcomes	Session 1	Session 1 (includes topic selection)
Module 2: Diabetes Basics	Session 2	Sessions 2–6 (patient choice)
Module 3: Problem-Solving and Talking to Your Doctor	Session 3	Sessions 2–6 (patient choice)
Module 4A: For General Diabetes Populations: Coping with Stress and Getting the Support You Need	Session 4 (staff choose 4A or 4B)	Sessions 2–6 (patient choice)
Module 4B: For Diabetes Populations with Severe and Persistent Mental Illness: Coping with stress, mental health conditions, and diabetes		Sessions 2–6 (patient choice)
Module 5: Nutrition and Healthy Eating	Session 5	Sessions 2–6 (patient choice)
Module 6: Lifestyle Change – Physical Activity, Sleep, and Good Habits	Session 6	Sessions 2–6 (patient choice)
Module 7: Follow-up Patient-Reported Outcomes, Reflection and Acknowledgment of Progress, Graduation		Session 6

*SMA* shared medical appointment, *TTIM* Targeted Training in Illness Management

#### Standardized diabetes SMA model distinguishing features

Standardized SMAs consist of the six-session TTIM curriculum delivered by health educators with general health coaching experience (e.g. a nurse, diabetes educator, or medical assistant). The TTIM standardized instructor’s manual specifies that the TTIM modules are delivered in a set order (session topics as listed in Table 2) and that care should be taken to adhere to the time schedule for each subtopic, to ensure all curriculum content is fully covered.

#### Patient-driven SMA model distinguishing features

Patient-driven SMAs consist of the six-session TTIM curriculum delivered by a multidisciplinary care team consisting of a health educator, a behavioral health provider, and a diabetes peer mentor. The health educator is the group facilitator for four TTIM sessions, while the behavioral health provider facilitates two sessions in their area of expertise (such as the problem solving and social skills, mental health, and general stress and coping modules). The peer mentor co-facilitates all visits and reinforces the curriculum by sharing their personal experience and perspective. Peer mentors are available to provide one-on-one support either in person or by telephone.

Patient-driven SMAs support patient selection of topic order. At the end of Session 1 (always module 1), patients select the order of modules 2–6 to cover in subsequent sessions for their cohort. The instructor’s manual provides guidance on this activity. Module 7 (debriefing and maintenance) is always covered last and combined with another module. During each session, the group facilitator(s) follows the lead of the patients in determining how much time is spent on each subtopic, rather than being expected to stick to the subtopic time schedule.

### Implementation framework and strategies

Implementation of SMA is guided by the Replicating Effective Programs (REP) framework [37]. The REP framework helps to guide study teams through the process of engaging practice implementation teams in packaging evidence-based interventions and refining study protocols to best align with practice priorities, workflows, resources, and preferences. The REP implementation process involves a pre-condition phase (e.g. packaging intervention for training and assessment using stakeholder input), a pre-implementation phase (e.g. orientation, explain core elements, customize delivery, logistics planning, staff training, and technical assistance), an implementation phase (e.g. ongoing support and partnership, booster training, fidelity monitoring), and a maintenance and evolution phase (e.g. understanding requirements for sustainability).

Consistent with Enhanced REP, Invested in Diabetes study practices receive the following implementation support: access to condition-specific TTIM materials on the study website; a one-day condition-specific training for any care team member who will deliver TTIM; a 1-h training for anyone who will serve as a prescribing provider; and 4–6 facilitation sessions with a practice coach. The coach helps practices create tailored workflows to prepare for and conduct SMAs, addresses logistical issues such as physical space for SMAs and prescribing provider visits, scheduling groups, and billing and reimbursement, helps plan strategies for identifying, recruiting, and retaining eligible patients, and serves as a liaison to the study team for data collection purposes. In patient-driven practices, coaches guide practices in selecting peer mentors; peer mentors are invited to participate in TTIM trainings with practice care team members, when possible, and are invited a 5-h peer mentor training. Practice representatives are invited to join quarterly condition-specific conference calls, as a learning community for sharing experiences and problem-solving around SMA delivery and sustainability.

### Outcomes and measures

The outcomes for this study are organized by the RE-AIM (Reach, Effectiveness, Adoption, Implementation, and Maintenance) framework [38, 39]. Table 3 shows a summary of outcomes, measures, and data sources corresponding to the RE-AIM dimensions.

**Table 3:** Summary of outcomes, measures, data sources and data collection timing for Invested in Diabetes

Outcome domain	Construct	Source	Metric/Measure	Timing
Patient reach	Service acceptance	Tracking spreadsheet	Attendance at initial SMA session among all invited	Initial SMA session
	Participation	Tracking spreadsheet	n/% and types of sessions attended	Monthly
		Interviews	Patient reasons for participation/non-participation	Within 3 weeks of last session
	Characteristics of participants	EHR	Demographics (age, gender, insurance, race/ethnicity) and clinical status (co-morbidity index [40]; insulin dependence, mental illness)	Collected during routine care
Patient-level effectiveness outcomes	Diabetes distress	Survey	DDS-17	1st and last SMA session
	Autonomy support	Survey	Health Care Climate Questionnaire	1st and last SMA session
	Perceived competence	Survey	Perceived Competence Scale	1st and last SMA session
	Self-care behaviors	Survey	Summary of Diabetes Self-Care Activities [41]	1st and last SMA session
	Health literacy (moderator)	Survey	Limited Health Literacy	1st SMA session
	Clinical outcomes	EHR	HbA1c, blood pressure, BMI	Collected during routine care (per 3–6 months)
	Patient experience and out-of-pocket costs	Interviews	Interview guide	≥ 3 weeks of last SMA session
Practice-level effectiveness	Team-based care	Survey	Relational coordination survey [42]	Baseline, midpoint, end of implementation
	Quality of care	Survey	Assessment of Chronic Illness Care [43]	Baseline, midpoint, end of implementation
Practice-level adoption, implementation, maintenance	Intervention fidelity and adaptations	Tracking spreadsheet, observation	Fidelity and adaptations observation guide	1 session observed per quarter per practice
	Practice culture	Survey	Practice Culture Assessment	Baseline, midpoint, end of implementation
	Practice motivations for adoption, perceived value and sustainability SMAs	Qualitative Interviews	Interview guide	Baseline, midpoint, end of implementation
	Implementation cost of SMAs	Survey	Time-Driven Activity-Based Costing framework [44]	Baseline, midpoint, end of implementation

*BMI* body mass index, *DDS-17* Diabetes Distress Scale-17, *EHR* Electronic Health Record, *SMA* shared medical appointment

#### Practice-level measures

Practice-level measures include practice context, which often influence efforts to improve diabetes care [45], including measures of relational coordination (using the Relational Coordination Survey [42], a measure of team-based care designed to measure relational coordination, communication, and relationships in particular work processes in primary care teams [46]), practice CCM-consistent care (using the Assessment of Chronic Illness Care [[ACIC]) [43], and practice culture (using the Practice Culture Assessment) [47]. Practice representatives complete measures of SMA resource requirements using a time-driven activity-based costing framework [44] to assess use of staff time, workflows, required materials and supplies, and other resources needed to deliver each SMA model, distinguishing between early and late implementation phases.

One-on-one, in-person key informant interviews [48] are conducted with practice members involved in SMAs at baseline, midpoint, and at the end to assess practice perceptions of the value and sustainability (burden, complexity, and potential for widespread uptake) of patient-driven and standardized diabetes SMAs. Baseline interviews focus on importance and interest in the upcoming SMAs, factors thought to affect adoption of the SMAs, and anticipated patient response to the SMAs. Mid- and endpoint interviews elicit the participant’s experiences with the SMAs, including a cognitive task analysis of the intervention as delivered in the practice, to provide a detailed understanding of fidelity and any possible adaptations, while illuminating gaps in understanding [49]. Final interviews specifically focus on recommendations for other practices and plans for continuing SMAs.

An observation guide was developed for this study to track fidelity and adaptations to intervention content (use of TTIM), to intervention delivery (care team members present, topic order and selection), and to facilitator style (reflection of SDT principles). Observers indicate topics covered and intervention delivery at observed sessions on a checklist and then rate facilitator style using open and closed ended items reflecting SDT principles (autonomy, competence, and relatedness support).

#### Patient-reported outcomes measures

Patient stakeholders selected diabetes distress as the primary patient-centered outcome, measured using the validated 17-item Diabetes Distress Scale (DDS-17) [50, 51]. Respondents indicate on a scale of 1–6 the extent to which they experience bothersome distress in four domains: emotional, regimen, interpersonal, and healthcare navigation burden. The DDS-17 has been demonstrated to be strongly related to and prospectively predictive of diabetes self-management behaviors and glycemic control and has discriminant validity from depression measures [10]. Perceived autonomy support and self-determination in healthcare settings (SDT constructs) are measured using the six-item Health Care Climate Questionnaire (HCCQ) and the four-item Perceived Confidence Scale (PCS) [52]. Diabetes self-management behaviors are measured with the Summary of Diabetes Self-Care Activities (SDSCA). This 11-item survey assesses self-reported dietary adherence, physical activity, and medication adherence, and is the most widely used and validated brief patient-reported scale for diabetes self-management behaviors [41]. All are valid and reliable self-report measures. Patient out-of-pocket costs are collected from responses to select survey questions adapted from the Medical Expenditure Panel Survey and the National Health Interview Survey [53]. Patient time commitments will be assessed from select items from the American Time Use Survey, which measures time spent receiving, waiting for, and traveling to receive medical services [54]. Health literacy, a potential moderator, is measured using the Limited Health Literacy Scale [55].

#### Patient-level clinical outcomes

Clinical outcomes data (HbA1c, blood pressure, BMI) will be extracted from participating practices’ EHRs. All encounter data (dates, locations, visits and provider types, diagnosis codes, lab results, and vital signs) will be measured from nine months before through nine months after each patient’s initial SMA visit. Diagnosis codes (ICD-9/10) will be used to compute a co-morbidity index [40] and to identify patients with a diagnosis of mental illness for moderator analysis. Medication data will be used to assess insulin dependence.

#### Patient reach and participation in SMAs

We will assess reach and participation of each SMA model using a spreadsheet maintained by practices tracking patients who agree to participate in SMAs versus actually attend and the number of sessions actually attended for each patient. Session attendance will be recorded by the SMA coordinator in each practice. Characteristics of those who participate (relative to the general patient population in each practice, based on practice characteristic surveys) will be described using EHR data on demographics and clinical status.

### Implementation timeline

**Fig. 2:**
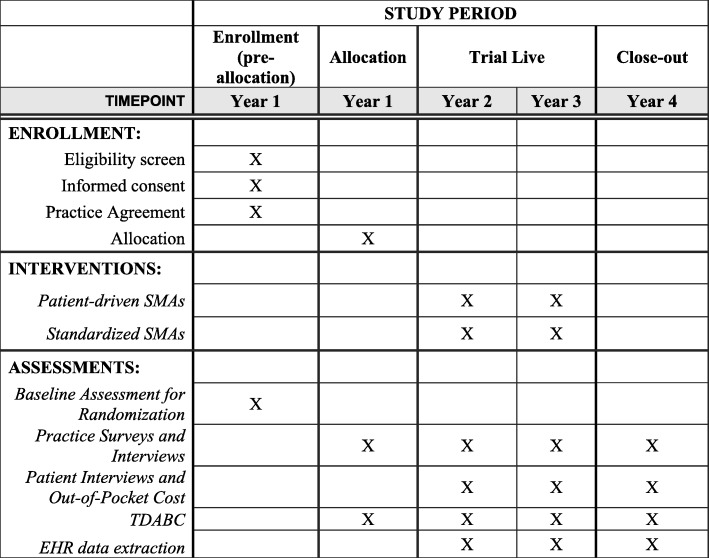
SPIRIT Figure for Invested in Diabetes project timeline

The anticipated timeline for practice participation is 37 months, including a four-month baseline data collection, training, and planning period, a 24-month active intervention period, and a nine-month follow-up period. Individual patient participation timelines will range from six weeks to six months, depending on practice preference for session frequency, plus additional time for select patients to complete interviews and additional surveys following SMA participation. In addition, practices will extract patient-level clinical data from EHRs from the nine months before and after each individual’s participation in SMAs. For the full timeline, see Fig. 2.

### Sample size

The planned sample size is 20 clinics and 1440 patients (10 clinics and 720 patients per condition), allowing for an attrition rate of approximately 15%–20% (leaving 600 patients per arm). We estimated the minimum effect sizes detectable for different power calculations for various numbers of practices and patient sample sizes and intraclass correlations (0.03 and 0.05), with effect sizes of approximately 0.27–0.33 for intent-to-treat analyses of primary outcomes with a type-1 error rate of 0.05. The sample size is powered for planned subgroup analyses for patient characteristics (e.g. mental illness co-morbidity, health literacy, insulin dependence) and for practice characteristics (FQHC vs private/commercial practice, urban vs rural). An effective sample size of 101 per subgroup is required to detect a medium linear trend effect between groups (increasing from 0 to 0.5 SD) using general linear mixed models with random slopes and intercepts [56].

### Recruitment

#### Practice recruitment

Practices are recruited through existing relationships with investigators and collaboration with Practice-Based Research Networks (PBRNs), including the State Networks for Ambulatory Practices and Partners (SNOCAP) in Colorado and the American Academy of Family Physicians National Research Network (AAFP NRN). PBRN member practices and other regional practices known to the study team to have an interest in implementing diabetes SMAs received email communications with a one-page description of the project. Those who respond to email communications were scheduled for additional phone calls and/or in-person meetings with the principal investigators to discuss the project requirements and incentives for participation. Each practice site receives $64,000 over the course of four years to support research activities (this does not cover clinical service delivery).

#### Provider and staff recruitment

Organizational leadership, providers, and other clinic staff are invited by the project study team to participate in practice surveys and interviews. The goal is to collect surveys from at least 70% of all practice staff and providers, and 100% of personnel who are (or will be) directly involved in diabetes SMA delivery.

#### Patient recruitment

SMAs are offered to patients through their regular primary care offices as a practice-level quality improvement initiative. Specifically, practices engage patients in treatment as they do in real-world care (i.e. not for research purposes) using reminder and follow-up calls to encourage attendance. Recruitment strategies vary by practice and can include identifying patients in existing diabetes registries, systematic screening and referral for new cases of T2DM, flyers posted in the practice, and provider-initiated referrals.

A subset of 3–5 SMA patients per practice will be recruited to participate in qualitative interviews and patient out-of-pocket cost and time commitment surveys. Participants from all classes will be reviewed by the SMA coordinator for potential interviewees. The SMA coordinator will use an opt-out procedure: mailing the participant a letter of invitation for the interview and survey and that they can opt out of participation. If the patient declines, s/he will not be contacted by the study team. If they accept, further recruitment will be done by the study team. Participants are compensated with a $50 gift card for completing the 60-min interview plus oral survey administration.

### Allocation

Covariate constrained randomization (CCR) is used to enhance internal validity and achieve balanced study arms in cluster randomized trials [29–31]. Before randomization, a representative for each participating practice completes a brief assessment to collect information for the CCR procedure. This information includes patient and practice characteristics that may systematically influence the practice’s ability to implement the intervention(s) or be associated with the outcome, such as being part of an affiliated health system, practice size, practice type (FQHC, private/system-affiliated practice), previous experience with SMAs, and presence of a quality improvement team. All possible combinations of two groups of eligible practices are generated using the SAS interactive matrix language procedure [57]. For each randomization, a balance criterion, defined as the sum of squared differences on standardized variables between arms, is computed. After examining the balance criterion distribution, an optimal set of randomizations is identified (best 5%–10%), from which one is chosen using a random number generator.

### Quantitative data collection

#### Practice-level outcomes

Practice surveys are administered on paper to practice clinicians and staff at baseline (pre-implementation state), approximate midpoint (early impressions), and approximate endpoint (final impressions) of the implementation phase. Fidelity and adaptations to SMA processes, content, and format are evaluated by study staff using a checklist for fidelity and adaptations monitoring. A randomly sampled 8%–10% of SMA sessions will be observed and coded for fidelity and adaptations to determine if sessions covered relevant TTIM topics, format was appropriate, if appropriate care team members were present, and if sufficient time was devoted to the summary and review portion.

#### Patient-reported outcomes and patient reach

Patient surveys will be completed during the first and last SMA sessions. The surveys are considered part of the intervention, as the TTIM script includes encouragement to have groups reflect upon the surveys to inform patient goal setting and topic selection (at session 1) and celebrate progress and improvement (at the end of session 6). The SMA coordinator will attempt to collect surveys for all patients, including those who miss the final session. Patient participation and survey data will be tracked by the SMA coordinator in a tracking spreadsheet. Patient out-of-pocket cost surveys will be administered separately for a subset of patients by a member of the study team.

#### Patient-level clinical outcomes

Clinical outcomes data for each patient will be extracted from participating practices’ EHRs for the period nine months before and after each patient’s initial SMA session. EHR extracts will be requested for all patients enrolled to date at the midpoint and endpoint of the implementation phase. Patients with diabetes are typically seen in primary care every 3–6 months; data on HbA1c, BMI, and blood pressure are collected routinely at these visits. Using data collected in the course of routine care is a pragmatic feature of this project, reducing costs and burden to practices and patients. As clinical outcomes will be gathered from practices’ EHRs, data availability will not depend on patient attendance at all six sessions, allowing robust estimates of comparative effectiveness of interventions in real-world contexts, in which patients vary in frequency of attendance.

The SMA coordinator will provide a list of participating patients to a practice data analyst, who will then pull requested data elements for participating patients following specifications provided by the research team. Data will be stripped of direct identifiers to create a limited dataset with a random unique patient identifier. Following data use agreements, data will be transferred to the research team using a secure cloud-based encrypted transfer mechanism, cleaned, and standardized across practices according to the Observational Medical Outcomes Partnership common data model [58]. Extraction specifications will be refined as needed after initial data review, per recommendations for data quality checking in comparative effectiveness research (e.g. assessing attribute domain constraints including ranges, relational integrity rules, historical data rules including temporal components, and missingness) [59].

### Qualitative data collection

#### Patient interviews

Interviews will be conducted using a semi-structured interview guide. Interviews will assess patient experience of SMAs specifically and diabetes care more generally. Probes will include the various elements of SMAs and which were most valuable (emphasizing exploration of the elements thought to reflect SDT constructs), reasons for participation or non-participation in SMAs, barriers and facilitators to participation, experience with care team members, and effects on self-management behaviors. To avoid contaminating the intervention, patients will be interviewed after their respective SMA is completed, including those who attended all sessions, as well as patients who prematurely discontinued, to better explore a range of participation experiences. Participants will be purposefully selected to reflect a variety of ages, race/ethnicity, and genders, and balanced across the two study arms (standardized and patient-driven SMAs). Three to five patient interviews per practice will be done, until thematic saturation is reached.

#### Practice interviews on perceptions of value and sustainability

One-on-one key informant interviews [48] will be conducted with 3–5 practice members involved in SMAs, per practice, at the beginning, approximate midpoint, and the endpoint of the implementation period. Interview guides will cover practice perceptions of the value and sustainability (burden, complexity, and potential for widespread uptake) of patient-driven and standardized diabetes SMAs. A semi-structured interview guide will be developed for each time period. Three to five interviews per practice per timepoint will be conducted, until thematic saturation is reached.

### Plans to promote participant retention and complete follow-up

Practices will engage patients in treatment as they would otherwise do in real-world care (i.e. not for research purposes), using reminder and follow-up calls to encourage patient attendance at visits. SMA coordinators will call patients to complete final patient-reported outcome measures if they are unable to attend the final session.

### Data management

All data will be stored on a secure password-protected server. Outside of the participating practices, individually identifiable health data will not be disclosed to the study team or anyone else. Specifically, random identifiers will be assigned to patients, which will be stored in the practices’ databases to allow linkage of clinical and survey data. All patient-level data will be stripped of direct identifiers before submission to the study team. The study team will not have access to contact information for potential participants unless they voluntarily provide this information (i.e. for patient interviews). Required Institutional Review Board (IRB) approvals and data use agreements among participating organizations have been obtained and study procedures approved by Colorado Multiple Institutional Review Board on 12 March 2018.

### Statistical methods

#### Missing data

We will examine the data carefully before analysis to determine whether patterns of missing are ignorable (Missing Completely At Random [MCAR] or Missing At Random [MAR]) or non-ignorable (Missing Not At Random [MNAR]) [60–63]. If ignorable, we will employ likelihood-based methods that utilize all available data, adjusting for covariates associated with missingness. If missingness is non-ignorable we will employ pattern mixture models [64]. Sensitivity analyses will be conducted using multiple imputation approaches.

#### Quantitative analysis

Descriptive statistics will first be computed for baseline patient and practice characteristics, followed by examining initial differences between: (1) intervention arms; and (2) patient dropouts versus non-dropouts. Patient-level covariates will be screened in bivariate analyses and included in multivariate analysis if related to outcomes at *p* < 0.2 or associated with dropout [65]. Covariates (to adjust for potential confounding) and potential moderators will include age, gender, race/ethnicity, co-morbidity index, insulin dependence, baseline diabetes distress, health literacy, and mental illness. For testing hypotheses I–IV (intervention arm differences in change in patient-reported outcomes, clinical outcomes, patient participation, and practice survey measures), we will employ intent-to-treat analyses using general (generalized) linear mixed models to incorporate data structures that are both hierarchical (by practice) and longitudinal (by time) [66–71]. Hypothesis tests will be two-sided with α = 0.05. All statistical analyses will be performed using SAS version 9.4 (SAS Institute Inc., Cary, NC, USA).

In recent literature on cluster randomized trials, general (or generalized) linear mixed models, adjusted for covariates are recommended for analysis of cluster randomized trials [72, 73], even after using such procedures as constrained randomization [74]. Likelihood based models using all available data are the preferred method for analyzing longitudinal data with dropout under Missing at Random (MAR) conditions [61, 75–77]. This will be our primary analysis; however, we will also examine change scores as outcomes, adjusting for baseline, in sensitivity analyses.

#### Patient cost/time and practice resource/time data analysis

Patient cost/time and practice resource and use of staff time data will be examined using simple descriptive measures, including range and means. Descriptive measures will be calculated for practices in each study arm, as well as for each type of practice and by level of patient participation.

#### Moderator analyses

We will conduct exploratory analyses to test for potential effect modification (moderator of intervention effectiveness) by selected patient characteristics. Mental illness co-morbidity is our primary target for moderator analyses and will be examined for each of the hypotheses. Additional sub-populations of interest are defined by gender, Hispanic ethnicity, and health literacy, as existing evidence suggests possible differential participation and effectiveness for these groups [78–80]. Due to the exploratory nature of these analyses, we do not plan to adjust for multiple comparisons in moderator analyses. However, interpretation of results will report on all subgroup analyses and take into account the number of subgroup analyses performed. These analyses will be adjusted for clustering.

#### Qualitative analysis

A qualitative analysis of practice and patient interview data will be conducted by 2–3 qualitative researchers with ongoing input and direction from the core study team. Interview data along with associated field notes and observations data will be transcribed, cleaned, and entered in the ATLAS.ti qualitative software program. For all analyses, we will begin with a grounded hermeneutic editing approach to help identify themes that are “grounded” or developed from an interpretation of the data [81]. The analyst team will determine the key themes and the associated definitions and labels (“codes”), which will be vetted with the study team and stakeholder representatives. Analysts will code the data using a coding and editing approach as outlined by Addison [81].

### Data monitoring

Oversight for data safety and monitoring of the randomized controlled trial portion of the study will be conducted by a researcher who is not involved in the project. Accordingly, a general internal medicine physician with experience in diabetes care, pragmatic trials, and protection of human participants, will serve as a Data Safety Monitor (DSM) for the trial. In this capacity, the DSM will provide independent observation and verification of protocol compliance, recruitment and study progress, and data completeness. This will be done through correspondence with the principal investigator and by reviewing draft annual reports on these parameters provided by the study team. The DSM will also monitor the study for adverse events and the study team’s response to these events, should any occur. A letter summarizing the DSM’s findings will be included in the finalized annual project reports for the funder. Though adverse events are not anticipated, should any occur they will be reported to all involved IRBs and the DSM at the time of the event; copies of all related correspondence with the IRBs and funder will be shared with the DSM.

### Harms

Risk of harm to participants is minimal. Should any occur, they will be reported to all involved IRBs and the DSM in accordance with federal and institutional policies. To mitigate risk of psychological discomfort and/or time burden, participants will be informed that they may choose not to complete any questions that make them uncomfortable and they may choose to withdraw from the study at any time without losing any benefits to which they may be entitled.

### Dissemination plans

We will disseminate findings via messages and strategies tailored to key audiences, who have different information needs, preferences, and perspectives regarding whether and how to offer or participate in diabetes SMAs. Study practices will disseminate results within their organizations and, in turn, these organizations will help disseminate results to patients and their communities, behavioral health and medical providers, health plans, and state and national professional organizations. We will prioritize engagement of stakeholders (patients, providers, and health plans) in the dissemination process, who will be invited to be co-authors on manuscripts, professional conference and community presentations, and in electronic media dissemination per community-based participatory research standards. The research team will also be available for consultation to other clinics seeking to implement the SMA models. We will conduct workshops at professional meetings frequented by our target audiences. We will also make resources available on our study website.

## Discussion

As a pragmatic trial, the Invested in Diabetes study is intended to be flexible in working with real-world practices that care for patients with diabetes. The protocol was refined based on practice and patient stakeholder input during the first year of the project, including identifying core intervention elements that should remain constant (i.e. the distinguishing features between SMAs), as well as opportunities for practices to adapt the intervention to their context and setting. For example, practices will be able to assign a broad array of healthcare team members to the health educator role for facilitating diabetes SMAs, such as nurses, diabetes educators, medical assistants, and others – so long as they were not behavioral health providers (a core element of the patient-driven condition). Tension between fidelity to core elements and adaptations is a common challenge in implementation studies, as practices often make changes without necessarily acknowledging such a change [82]. As a result, it is important to track both fidelity and adaptations (using the methods described in this protocol) and describe protocol deviations to inform generalizability of findings.

As with any major practice change, implementing diabetes SMAs is expected to incur practical and operational challenges. Thus, SMA implementation will be guided by an enhanced version of the REP framework [37]. The use of implementation strategies described by REP will be used to ensure practices maintain fidelity to intervention protocols while supporting appropriate adaptation to their unique needs and resources. By packaging the study and curriculum to align with practice needs and interests, and providing practice facilitation to support practice change, implementation of SMAs should be done faithfully to the protocol – helping ensure that resulting findings reflect a true test of the SMA features under investigation.

Finally, the Invested in Diabetes study was developed in collaboration with patients with diabetes, their care partners, and clinicians from participating practices. Research questions, outcomes, and intervention content were selected by these stakeholders, so that resulting findings may better inform clinical and operational decisions for healthcare professionals treating patients with diabetes. A robust mixed methods evaluation will seek to thoroughly confirm or refute study hypotheses, while providing elaborative detail. In conducting this study, we hope to inform future care models for the many individuals who have diabetes, helping them better achieve diabetes control, reduce diabetes distress, and increase both longevity and quality of life.

## Trial status

This manuscript describes version 2 of our protocol, last updated 7 March 2019. Enrollment to the study began in January 2019, with all practices enrolling patients by August 2019. Anticipated end to patient enrollment is December 2020.

## Supplementary information

**Additional file 1.** SPIRIT 2013 Checklist: Recommended items to address in a clinical trial protocol and related documents.

## Data Availability

Not applicable.

## Abbreviations

ACIC: Assessment of Chronic Illness Care
CCM: Chronic Care Model
DDS-17: Diabetes Distress Scale
DSM: Data Safety Monitor
DSME: Diabetes Self-Management Education
EHR: Electronic Health Records
FQHCs: Federally Qualified Health Centers
GLMM: Generalized Linear Mixed Model
IRB: Institutional Review Board
RE-AIM: Reach, Effectiveness, Adoption, Implementation, and Maintenance
REP: Replicating Effective Programs
SDSCA: Summary of Diabetes Self-Care Activities
SDT: Self-Determination Theory
SMAs: Shared Medical Appointments
T2DM: Type 2 diabetes mellitus
TDABC: Time-Driven Activity-Based Costing
TTIM: Targeted Training for Illness Management
