# Correction to: The Invested in Diabetes Study Protocol: a cluster randomized pragmatic trial comparing standardized and patient-driven diabetes shared medical appointments

**DOI:** 10.1186/s13063-020-4110-0

**Published:** 2020-02-18

**Authors:** Bethany M. Kwan, L. Miriam Dickinson, Russell E. Glasgow, Martha Sajatovic, Mark Gritz, Jodi Summers Holtrop, Don E. Nease, Natalie Ritchie, Andrea Nederveld, Dennis Gurfinkel, Jeanette A. Waxmonsky

**Affiliations:** 1 University of Colorado School of Medicine, 13199 E Montview Blvd Ste 210, Aurora, CO 80045 USA; 2 VA Eastern Colorado QUERI and Geriatric Research Centers, 1055 Clermont St, Denver, CO 80220 USA; 3 Case Western Reserve University, 10900 Euclid Ave, Cleveland, OH 44106 USA; 4 Denver Health and Hospital Authority, 777 Bannock St, Denver, CO 80204 USA

**Correction to: Trials**

**https://doi.org/10.1186/s13063-019-3938-7**

After publication of our article [1] the authors have notified us that the title for Figure 1 was incorrectly captioned in addition it was noticed that the Interventions section contained two errors.

- Originally published Figure 1 title:
  SPIRIT Figure for Invested in Diabetes project timeline
- Correct Figure 1 title:
  Invested in Diabetes study conceptual model.

The error: Patients step out of group for brief [5–10]individual visits with a provider with prescribing privileges, who provides medication management, orders and referrals, and patient-specific medical advice, i.e. curriculum, dose of intervention, frequency of sessions, visits with medical providers, and group size are consistent across study arms, and are thus not variables in the study.

Should instead read: Patients step out of group for brief (5–10 minutes) individual visits with a provider with prescribing privileges, who provides medication management, orders and referrals, and patient-specific medical advice. Curriculum, dose of intervention, frequency of sessions, visits with medical providers, and group size are consistent across study arms, and are thus not variables in the study.

The original article has been corrected.
